# RNA-Sequencing-Based Transcriptomic Analysis Reveals a Role for Annexin-A1 in Classical and Influenza A Virus-Induced Autophagy

**DOI:** 10.3390/cells9061399

**Published:** 2020-06-04

**Authors:** Jianzhou Cui, Dhakshayini Morgan, Dao Han Cheng, Sok Lin Foo, Gracemary L. R. Yap, Patrick B. Ampomah, Suruchi Arora, Karishma Sachaphibulkij, Balamurugan Periaswamy, Anna-Marie Fairhurst, Paola Florez De Sessions, Lina H. K. Lim

**Affiliations:** 1 Department of Physiology, Yong Loo Lin School of Medicine, National University of Singapore, Singapore 117456, Singapore; jianzhou.cui@nus.edu.sg (J.C.); dhakshayinikcmorgan@gmail.com (D.M.); chengdaohan@u.nus.edu (D.H.C.); slin.foo@u.nus.edu (S.L.F.); gracemaryyap@u.nus.edu (G.L.R.Y.); pba2113@cumc.columbia.edu (P.B.A.); suruchi.arora1987@gmail.com (S.A.); phsksb@nus.edu.sg (K.S.); 2 Immunology Program, Life Sciences Institute, National University of Singapore, Singapore 117456, Singapore; 3 Graduate School for Integrative Sciences and Engineering, National University of Singapore, Singapore 119077, Singapore; 4 GIS Efficient Rapid Microbial Sequencing (GERMS), Genome Institute of Singapore, Agency for Science, Technology and Research (ASTAR), Singapore 138672, Singapore; bperiaswamy@ibn.a-star.edu.sg (B.P.); florezdesessions@gmail.com (P.F.D.S.); 5 Institute of Molecular and Cell Biology (IMCB), Agency for Science, Technology and Research (ASTAR), Singapore 138673, Singapore; annamarie@imcb.a-star.edu.sg

**Keywords:** influenza, RNA-sequencing, transcriptomics, autophagy, Annexin-A1

## Abstract

Influenza viruses have been shown to use autophagy for their survival. However, the proteins and mechanisms involved in the autophagic process triggered by the influenza virus are unclear. Annexin-A1 (ANXA1) is an immunomodulatory protein involved in the regulation of the immune response and Influenza A virus (IAV) replication. In this study, using clustered regularly interspaced short palindromic repeats (CRISPR)-Cas9 (CRISPR associated protein 9) deletion of ANXA1, combined with the next-generation sequencing, we systematically analyzed the critical role of ANXA1 in IAV infection as well as the detailed processes governing IAV infection, such as macroautophagy. A number of differentially expressed genes were uniquely expressed in influenza A virus-infected A549 parental cells and A549 ∆ANXA1 cells, which were enriched in the immune system and infection-related pathways. Gene ontology and the Kyoto Encyclopedia of Genes and Genomes (KEGG) pathway revealed the role of ANXA1 in autophagy. To validate this, the effect of mechanistic target of rapamycin (mTOR) inhibitors, starvation and influenza infection on autophagy was determined, and our results demonstrate that ANXA1 enhances autophagy induced by conventional autophagy inducers and influenza virus. These results will help us to understand the underlying mechanisms of IAV infection and provide a potential therapeutic target for restricting influenza viral replication and infection.

## 1. Introduction

Influenza A virus (IAV) is a respiratory pathogen which causes widespread infections globally. It is made up of an enveloped capsid that encloses single-stranded RNA. The RNA genome encodes for 13 proteins [1]. Specific viral proteins such as hemagglutinin (HA) and neuraminidase (NA) [2] are found on the surface as antigenic glycoproteins, while others such as matrix1 (M1) and matrix2 (M2) are found on the inside of bilayer lipid membranes [3]. Another viral protein, non-structural protein 1 (NS1), inhibits type 1 interferon (IFN) synthesis and Double-strand RNA (dsRNA)dependent protein kinase R, and thus impedes the host innate response [4,5]. When the virus enters the host cell, the viral life cycle begins. It uses host cell machinery for its replication and transcription [6].

Macroautophagy, microautophagy and chaperone-mediated autophagy are the three different types of pathways involved in autophagy, and all of them require lysosomal degradation [7]. Autophagy plays a role in host defense during pathogenic invasions by preventing viral replication through the removal of pathogenic protein aggregates in the cytoplasm [8]. However, emerging evidence has demonstrated that the autophagy process is regulated by the influenza virus for its benefit [9]. During virus infection, the virus requires the host cells to survive and proliferate and thus it will activate pro-survival mechanisms that include autophagy. Similarly, IAV diverts cell death induced by apoptosis to that of autophagy and this results in prolonged survival and increases virus titers because of enhanced viral replication and deregulation of immune responses [10]. The viral proteins, HA, M2 and NS1, have been reported to be involved in the induction of autophagy by IAV [11]. IAV can inhibit mechanistic target of rapamycin (mTOR) via regulating the mTOR inhibitor tumor suppressor protein 2 (TSC2) [12] and prevents autophagosome fusion with lysosomes via M2 [3], which contains a Microtubule-associated protein light chain 3 (LC3)-interacting domain, causing LC3 to localize to the plasma membrane [13]. This subverted autophagy as fusion was disallowed. Hence, influenza virus triggers the initiation of autophagy but prevents the final steps of autophagosome fusion with lysosomes, utilizing autophagy to accumulate viral components [14]. 

Briefly, macroautophagy begins with the isolation of the membrane to form a phagophore that is mediated by the unc-51 like autophagy activating kinase 1 (ULK1) kinase complex. Next, Beclin-1 and VSP34, also known as Class III phosphoinositide 3-kinase (PI3K) complex, drives nucleation of the isolated membrane. ATG9 and VMP1 subsequently recruit lipids to the isolated membrane. For the closure of isolated membrane and formation of an autophagosome, the two ubiquitin-like conjugate systems, ATG12-ATG5 and Microtubule-associated protein 1A/1B-light chain 3 (LC3), are involved. In the ATG12-ATG5 system, ATG7 (E1-like enzyme) and ATG10 (E2-like enzyme) are needed to help in the conjugation of ATG12 to ATG5, which is then linked to ATG16. Together the ATG12-ATG5-ATG16 complex forms an E3-like ligase of LC3 and stabilizes the autophagosome [15]. In the LC3 conjugation system, LC3 is cleaved by ATG4 to become LC3-I and LC3-I is converted to LC3-II after being conjugated to phosphatidylethanolamine. Hence, the ATG8/LC3 system plays an essential role in the proper development of autophagic isolation membranes and the biochemical changes of ATG8/LC3 (lipidation and membrane translocation) have been well established as an essential autophagy marker [16]. 

Annexin 1 (ANXA1), the 37 kDa protein containing 346 amino acids, was the first identified member of the annexin superfamily. It has a structure which consists of a core domain made of alpha helices. The regulatory region is localized at the *N* terminus, which contains sites for phosphorylation and proteolysis [17]. In the presence of calcium, ANXA1 binding to negatively charged phospholipids was mediated by Ca^2+^-binding motif which located in core domain [18]. ANXA1 was discovered to mediate the anti-inflammatory effect of glucocorticoids, where it inhibits the action of phospholipase A2 (PLA2) by both direct enzyme inhibition and suppression of cytokine-induced activation of the enzyme, limiting the supply of arachidonic acids needed for the synthesis of prostaglandins, thus suppressing inflammation [19]. Although initially discovered in the late 1970s due to its role in inflammation, ANXA1 has also been found to play a role in tumorigenesis, with multiple functions in proliferation, differentiation, apoptosis, migration and invasion [20]. We have recently reported that ANXA1 enhances endosomal trafficking of influenza virus and enhances apoptosis [21]. Furthermore, expression levels of ANXA1 were increased in porcine monocytes during infection with swine flu virus [22], and in human nasal swabs of influenza A virus-infected patients [21]. 

In this study, we aimed to understand the role of ANXA1 in influenza virus infection more deeply using RNA-sequencing based transcriptomic analysis. Using differential gene expression and gene ontology and subsequent verification experiments, we identified that ANXA1 plays an important role in autophagy induced by classical and viral means. 

## 2. Materials and Methods

### 2.1. Mice 

BALB/c ANXA1^−/−^ mice were a kind gift from Prof. Roderick Flower from the William Harvey Research Institute, UK. Mice were age-matched and BALB/c mice were used as control mice for each experiment. All mice were maintained under pathogen-free conditions in the animal housing unit and were transferred to the ABSL2 facility for experiments involving infection with IAV. All animal work was approved by the Institutional Animal Care and Use Committee (Protocol number R13-5101) and followed National Advisory Committee for Laboratory Animals Research (NACLAR) Guidelines on the Care and Use of Animals for Scientific Purposes.

### 2.2. Viruses

For viral propagation, 1 hemagglutinating unit (HAU) virus A/Puerto Rico/8/1934(H1N1) Influenza A virus (A/PR8) was injected into 10–12-day incubated chicken eggs and further incubated for 3 days. On day 3, the eggs were chilled at −80 °C to euthanize the embryo and the allantoic fluid was collected. The fluid was spun in 100,000 molecular weight cutoff (MWCO) concentrators to concentrate the virus and viral plaque assays were performed to quantify the viral titers before use.

### 2.3. Cell Culture 

The human epithelial lung cancer cell line, A549 parental cell line (CCL-185, ATCC, Gaithersburg, MD, USA), A549 ∆ANXA1 cells, ATG5 wild-type Mouse Embryonic Fibroblasts (MEFs) and ATG5^−/−^ MEFs were cultured at 37 °C in a humidified atmosphere with 5% CO_2_ incubator. The media used for full nutrient and starvation medium were Dulbecco’s Modified Eagle Medium (DMEM) and Earle’s Balanced Salt Solution (EBSS) respectively. 

### 2.4. A549-ΔANXA1 Cell Line Generation

To produce the A549-ΔANXA1 cell line, clustered regularly interspaced short palindromic repeat-caspase 9 (CRISPR-Cas9) transfection was performed. The plasmid contains the two ANXA1 single guide RNA (sgRNA) (pls2#-CAAACTGTGAAGTCATCCAA and pls4#-ATGCAAGGCAGCGACATCCG were generated by Horizon Discovery Group). The single cell line of A549-ΔANXA1 was isolated using the protocol from the Horizon Company online manual. Generally, A549 cells were seeded in 10 cm dishes and transfected with 10 μg of plasmid per dish using Turbofect, according to the manufacturer’s instructions. After 24 h, cells were sorted for positive green fluorescent protein (GFP) expression using the Beckman-Coulter Mo-Flo Legacy Cell Sorter into 96-well plates with 5 cells seeded per well. Cells were expanded to 6-well plates and a preliminary Western blot was conducted to screen for cells with less or no ANXA1 present compared to A549 control cells. Those with less or no ANXA1 present were then seeded again into 96-well plates as single clones and expanded. A secondary Western blot was conducted to determine single cell clones with no ANXA1 present.

### 2.5. Total RNA Extraction, Library Construction and RNA-Sequencing and Data Analysis

Total RNA was isolated from cells, post treatment, using the RNeasy mini column purification kit (Qiagen, Limburg, The Netherlands) according to the manufacturer’s instructions. Cells were washed in 1X PBS on ice, prior to RNA extraction. Total RNA extracts were run on the Agilent bioanalyzer using the eukaryote total RNA pico chips to determine the RNA integrity values (7.4–9.4, with an average of 8.8). TruSeq Stranded mRNA sample preparation was used as per the manufacturer’s instructions for next-generation library preparation. Briefly, library preparation entailed: Purification of mRNA using poly-T oligo-attached magnetic beads, fragmentation of mRNA, first and second strand cDNA synthesis, A-tailing and ligation of adapters with multiplex indexes, according to the manufacturer’s instructions. Samples were enriched with 15 PCR cycles followed by Agencourt AMPure XP magnetic bead (Beckman Coulter, Brea, CA, USA) clean up according to manufacturer’s instructions. Quality of cDNA libraries was checked with Agilent D1000 Tapestation Assay (Agilent 4200 Tapestation System). Next-generation sequencing was performed using Illumina Hiseq 4000 flow cell, 2 × 151 base pair-end runs. PhiX was used as control. 

The RNA-Seq transcriptome datasets were mapped to the Genome Reference Consortium Human Build 38 release 86 (GRCh38.r86) by using the Spliced Transcripts Alignment to a Reference (STAR) aligner [23]. Reads that were unambiguously mapped to a given gene were counted using the high-throughput sequencing (HTSEQ)-COUNT tool, available under the HTSeq python framework [24]. These gene-based raw read counts were used for differential gene expression analyses using Bioconductor package EdgeR [25]. Initially, sample read counts were adjusted for library size and normalized using the trimmed mean of m-values (TMM) method. Differential gene expression between groups were assessed using the exactTest method in EdgeR. Genes were called differentially expressed at false discovery rate (FDR) < 0.05. Differentially expressed genes with at least a log2 fold-change of +/− 1 were considered for further functional annotation.

RNA-Sequencing data was submitted to Sequence Read Archive (SRA). Accession number has been provided (BioProject ID: PRJNA637807). 

### 2.6. Gene Ontology (GO) and Gene Set Enrichment Analysis (GSEA) Pathway Analysis

Gene ontology (GO) enrichment analysis were performed by the Database for Annotation, Visualization and Integration Discovery (DAVID) Functional Annotation (https://david.ncifcrf.gov/home.jsp) and biological pathway analyses were carried out using REACTOME (http://www.reactome.org/PathwayBrowser). The differentially expressed genes (DEGs) list from two treatment groups: (1) A549 parental cells with or without infection (Group A: wild type (WT) infected versus WT Control) and (2) A549 ∆ANXA1 cells with or without infection (Group B: knockout (KO) infected versus KO Control) were selected to perform the GSEA analysis by using WebGestalt (http://www.webgestalt.org/option.php).

### 2.7. Western Blot 

Cells were harvested and centrifuged, and supernatant was collected for protein concentration using a BioRad spectrophotometer or stored at −80 °C. Samples were loaded onto 15% (sodium dodecyl sulphate-polyacrylamide gel electrophoresis) (SDS-PAGE) gel which was run in 1× running buffer at 120 V for 2 h following the transfer procedure. After which the nitrocellulose membranes were blocked using 3% skimmed milk before primary antibodies were added, and the blots were incubated overnight at 4 °C shaking. The secondary antibodies were added and were washed prior to chemiluminescent detection. The primary antibodies used were ANXA1 (sc-12740, Santa Cruz, Dallas, TX, USA), LC3 (# M152-3, MBL, Woburn, MA, USA), Atg3 (#3415, Cell signaling, Danvers, MA USA), β-Actin (#4970, Cell signaling, Danvers, MA, USA) and Glyceraldehyde 3-phosphate dehydrogenase (GAPDH) (#5174, Cell signaling, Danvers, MA, USA). The secondary antibodies used were goat anti-mouse Horseradish Peroxidase (HRP) (#sc-2005, Santa Cruz, Dallas, TX, USA) and goat anti-rabbit HRP (#sc-2030, Santa Cruz, Dallas, TX, USA).

### 2.8. Confocal Microscopy 

Coverslips were placed into each well of a 12-well plate. Cells were plated on top of the cover slips and left to incubate with full media and 10 uL of anti-LC3 B GFP antibody (Molecular probes by Life Technologies, Waltham, MA, USA) for 1 h at room temperature and treated with EBSS over various time points. 4% paraformaldehyde (PFA) was added to each well at 4 °C and DAPI (4′,6-diamidino-2-phenylindole) was counterstained for 10 min. The cover slips were washed with PBS 3x and mounted onto microscope slides. The dry slides were visualized with a confocal microscope (Leica LSM 510, Wetzlar, Germany). 

### 2.9. Influenza A Virus (IAV) Infection

For in vitro experiments, cells were plated in full media overnight, and washed with PBS. The inoculum containing 1 MOI (Multiplicity of infection) of HIN1 virus in serum-free medium was incubated with the cells according to the various treatment timings. After which, the media was aspirated, and the cells were washed with PBS. For in vivo experiments, WT and ANXA1^−/−^ Balb/c mice were infected with 500 plaque-forming unit (pfu) of influenza virus intratracheally. On days 0, 1, 3 and 5 post-infection, the mice were sacrificed, and their lung lysates were obtained for Western blot analysis.

### 2.10. Statistical Analysis

Data shown are the mean ±standard error of the mean (SEM) of 3–5 independent experiments for in vitro work and 3–5 mice for mice work. Student’s unpaired T test and two-way analysis of variance (ANOVA) for grouped data with Bonferonni’s multiple comparison tests were performed for all datasets. GraphPad Prism (GraphPad software, San Diego, CA, USA) was used for analysis.

## 3. Results

### 3.1. Detection of CRISPR-Cas9-Induced ANXA1 Mutations in A549 ∆ANXA1 Cells

First, to study the role of ANXA1 in the context of IAV infection, ANXA1 was deleted from A549 cells using Crispr-Cas9 technology. Two gRNA sequences that target ANXA1 were custom designed by and obtained from Horizon Discovery. The plasmid-encoding gRNAs that target ANXA1 also encode for GFP, which is expressed once the plasmid has been transfected into the cell. The plasmids were transfected into A549 cells separately and cells positive for GFP were sorted using the Beckman-Coulter Mo-Flo Legacy Cell Sorter into 96-well plates. Single cell clones were expanded, and validation experiments were performed to confirm the deletion. Mutation of ANXA1 in sgRNA #4 regions located in domain I introduced a frameshift mutation which translated into a truncated ANXA1 protein. The off-target effects of #4 sgRNA were also analyzed by online tool, COSMID (https://crispr.bme.gatech.edu/) [26] (Supplemental Table S1). Our sequencing results revealed that no off-target mutations were detected at any of the off-target sites.

#### Transcript Expression Profiling in Influenza Virus-Infected A549 Parental and ∆ANXA1 Cells

To understand the global and general transcripts expression profiles after IAV replication, and the importance of ANXA1 in this process, we performed global RNA-Sequencing (RNA-Seq) in A549 parental and A549 ∆ANXA1 cells with and without IAV infection: (1) A549 parental cells with or without infection (Group A: WT infected versus WT Control) and (2) A549 ∆ANXA1 cells with or without infection (Group B: KO infected versus KO Control). After normalizing read count data and setting the significance and fold-change (*p* < 0.01 and log2 fold-change ≥ |1|), transcripts were significantly changed or differentially expressed (DE) and are listed in the Supplemental Table S2. 

**Figure 1:**
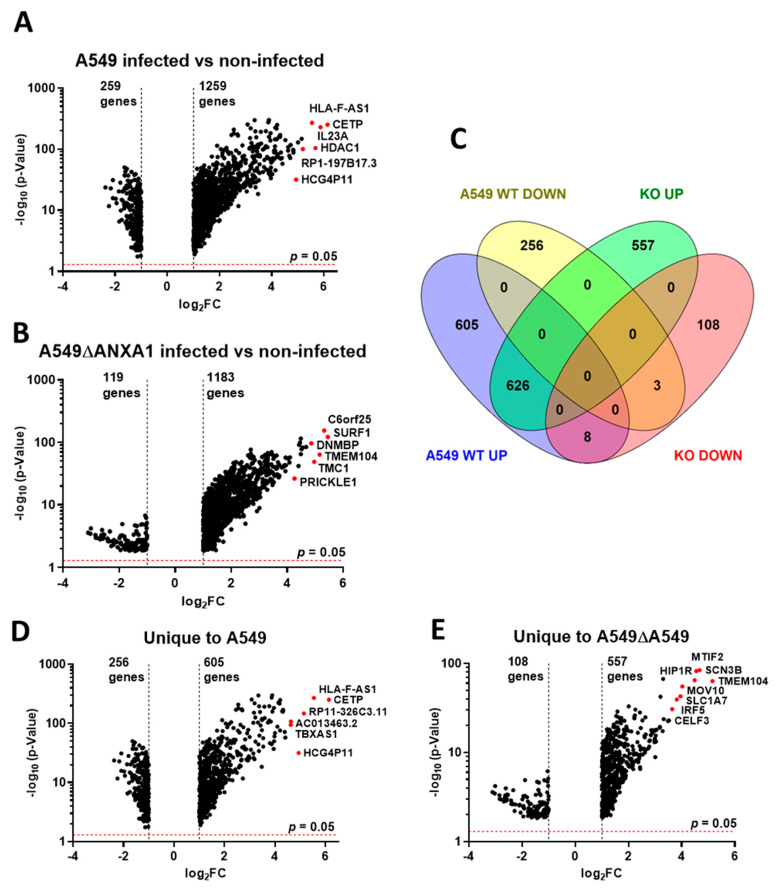
Global overview of the RNA-Sequencing data of influenza A virus (IAV)-infected lung epithelial (A549) cells with or without ANXA1 deficiency. (**A**,**B**) Volcano map showing the significant differentially expressed transcripts in A549 and A549-∆ANXA1. (**C**) The distribution abundance of differentially expressed genes/transcripts in upregulated and downregulated pattern in group A and B. MTBP (MDM2 Binding Protein), PMCH (Pro-Melanin Concentrating Hormone), FTO (alpha-ketoglutarate-dependent dioxygenase). (**D**,**E**) The unique expressed transcripts in group A and group B are plotted on a volcano map. The top genes selected showing the highest fold-changes are presented as red dots.

When A549-infected versus A549 control samples were compared, a total of 1498 differentially expressed transcripts were detected, of which included 1239 transcripts (82.7%) that were significantly upregulated and 259 transcripts (17.3%) which were significantly downregulated (*p* < 0.05) (Figure 1A). A549-ΔANXA1-infected cells were compared against A549-ΔANXA1-uninfected controls, and a total of 1302 differentially expressed transcripts were detected, and globally significant transcript expression patterns were plotted on a volcano map, illustrating that 90.9% of transcripts were upregulated while the remaining 9.1% were downregulated (Figure 1B). Three gene transcripts, MDM2 Binding Protein (MTBP), Pro-Melanin Concentrating Hormone (PMCH) and Fat mass and obesity-associated (FTO) were downregulated both in A549 parental and A549-∆ANXA1 cells, together with 626 other transcripts which were upregulated in both cell types, indicating that these transcripts are ANXA1-independent (Figure 1C). Two sets of transcripts which are uniquely expressed in A549-infected and A549-ΔANXA1-infected cells are listed in Supplemental Table S2 and globally expressed on volcano plots in Figure 1D,E. 605 upregulated transcripts and 256 downregulated transcripts were uniquely expressed in parental A549 cells infected with IAV, while 557 upregulated transcripts and 108 downregulated transcripts were uniquely expressed in A549-ΔANXA1 cells infected with IAV.

### 3.2. Gene Ontology (GO) and GSEA Pathway Analysis

**Figure 2:**
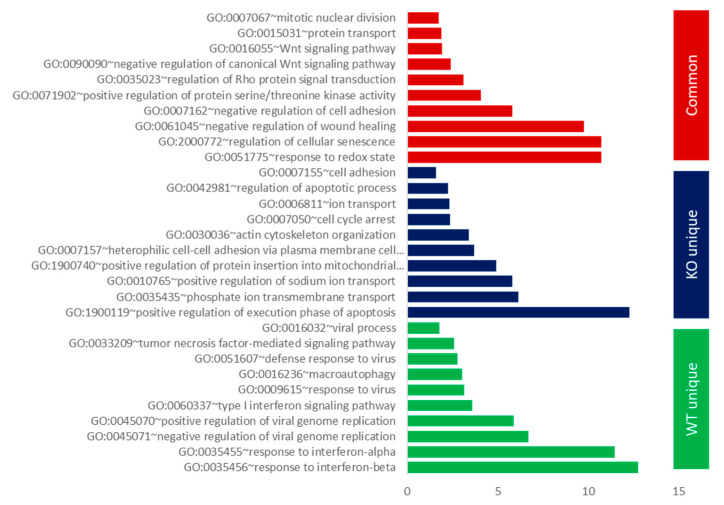
Gene ontology (GO) analysis of uniquely expressed genes/transcripts in A549 (WT) and A549-ΔANXA1 (KO) infected versus control samples and commonly expressed transcripts. Top 10 of the most highly enriched items in the categories of Biological process (BP) are presented in three groups, respectively (*p* < 0.01). Logarithm (base 2) of the odds ratio of the enrichment of the GO items. The larger this number is, the stronger the enrichment of the GO items among transcripts in the dataset.

The transcripts which were commonly expressed in both WT (A549 parental cells) and KO (A549-ΔANXA1) infected cells, as well as the transcripts which were uniquely expressed in both cell types, were analyzed further with gene ontology, focusing on biological process pathways. The top enriched fractions are shown in Figure 2 (*p* < 0.05) and Supplemental Table S3. For commonly expressed transcripts (red bars), representing ANXA1-independent genes, infection of IAV resulted in a high enrichment of the biological processes relating to the response to Redox state (GO0051775), regulation of cellular senescence (GO2000772) and negative regulation of wound healing (GO0061045). In unique transcripts, expressed uniquely in the A549 parental infected cells and not the A549-ΔANXA1 cells (blue bars), representing genes which require ANXA1 either to be expressed or repressed, the most enriched terms are the response to type-1 interferons (GO0035455/6), and the regulation of viral genome replication (GO045070/1), which we have shown previously to be regulated by ANXA1 [21]. Of interest, macroautophagy (GO0016236) is also a biological process which is highly enriched and requires ANXA1. 

For unique transcripts which are expressed in A549-ΔANXA1-infected cells and not A549 parental cells (green bars), representing genes which can be regulated by ANXA1, the enriched biological processes include positive regulation of apoptosis (GO1900119), regulation of mitochondrial apoptosis (GO1900740) and actin cytoskeleton organization (GO0030036), which have also been shown previously to be regulated by ANXA1 [21]. The global distribution pattern for the top enriched pathway in each group is presented in Supplementary Figure S1.

**Figure 3:**
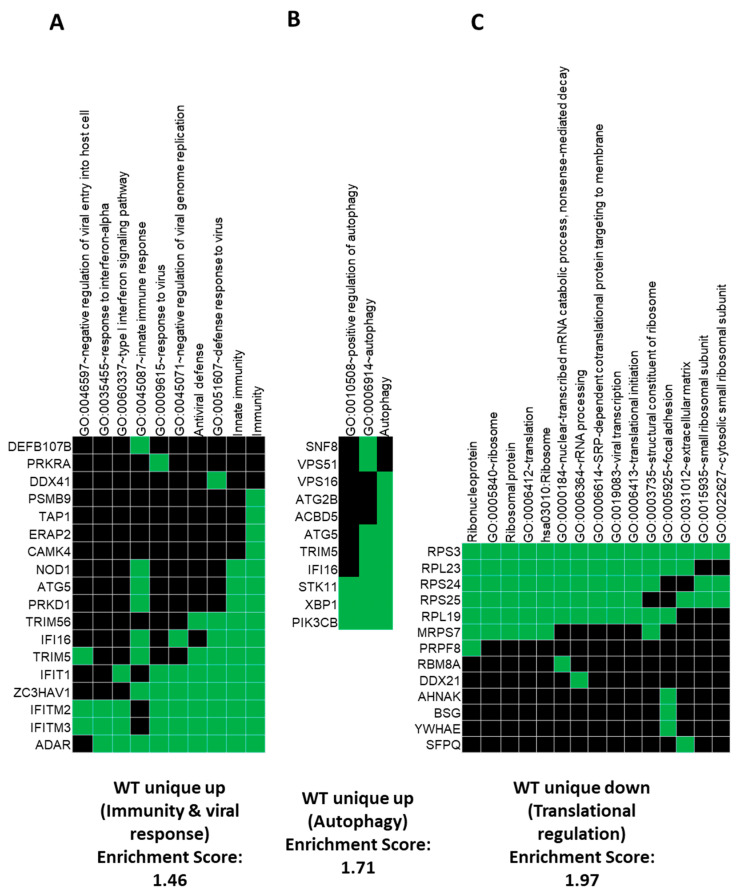
The specific genes involved in given gene ontology for uniquely expressed up and down genes in A549 (WT) and A549-ΔANXA1 (KO) infected versus control samples. Specific genes are highly enriched and involved in immunity and viral responses (**A**), autophagy (**B**) and translational regulation (**C**).

Using DAVID (https://david.ncifcrf.gov/home.jsp), we looked closer into the genes which are involved in immunity and viral responses in both cell types (Figure 3A), and Interferon Induced Protein With Tetratricopeptide Repeats 1(IFIT1), IFIT2 and IFIT3, (also known as interferon stimulated gene factor 56 (ISG56), ISG54 and ISG60, respectively), which are all interferon-induced anti-viral proteins, are increased in A549 WT but not A549-∆ANXA1 cells, signifying that ANXA1 is required for these genes. In addition, Tripartite motif-containing protein 5 (TRIM5) and TRIM56, which are tripartite motif-containing Ring-type E3-ubiquitin protein ligases, which are also anti-viral proteins, also require ANXA1 to be expressed. Autophagy-related proteins’ expression, which are controlled by ANXA1, include ATG5, ATG2B and other genes involved in protein trafficking in endosomes and lysosomes, such as vacuolar protein sorting 51 (VPS51) and VPS16 (Figure 3B). This is interesting as we have previously shown that ANXA1 can enhance endosomal trafficking of the virus [21]. Transcription factor XBP1 and serine threonine kinase STK11, which are also dependent on ANXA1 for expression after influenza infection, have both been shown to be important in the regulation of autophagy [27,28,29,30]. Therefore, we validated this finding in vitro in lung epithelial cells.

To further identify the potential function and pathway enrichment of DEGs in the two groups, GSEA was conducted to search significant pathways enriched in the highly expressed DEGs in both upregulation and downregulation genes (Supplementary Table S2). The top ten enriched gene sets in both up- and down-regulated expression pattern for Group A (WT infected versus WT Control) are shown in Supplementary Figure S2B. Two of the gene sets, i.e., plasma lipoprotein assembly, remodeling, and clearance, and Transcriptional Regulation by TP53 were enriched in upregulated DEGs (FDR > 0.05), while Viral mRNA Translation and Influenza Viral RNA Transcription and Replication were enriched in downregulated DEGs (FDR < 0.05) in group A (Supplementary Figure S2A). As for group B (KO infected versus KO Control), we found that gene sets of Regulation of Hypoxia-inducible Factor (HIF) by oxygen and Infectious disease were enriched in upregulated DEGs (FDR > 0.05), while Fc epsilon receptor (FCERI) signaling and Transport to the Golgi and subsequent modification were enriched in downregulated DEGs (FDR < 0.05) (Supplementary Figure S2B).

### 3.3. IAV Enhances Autophagy through Regulation of Autophagic Proteins

**Figure 4:**
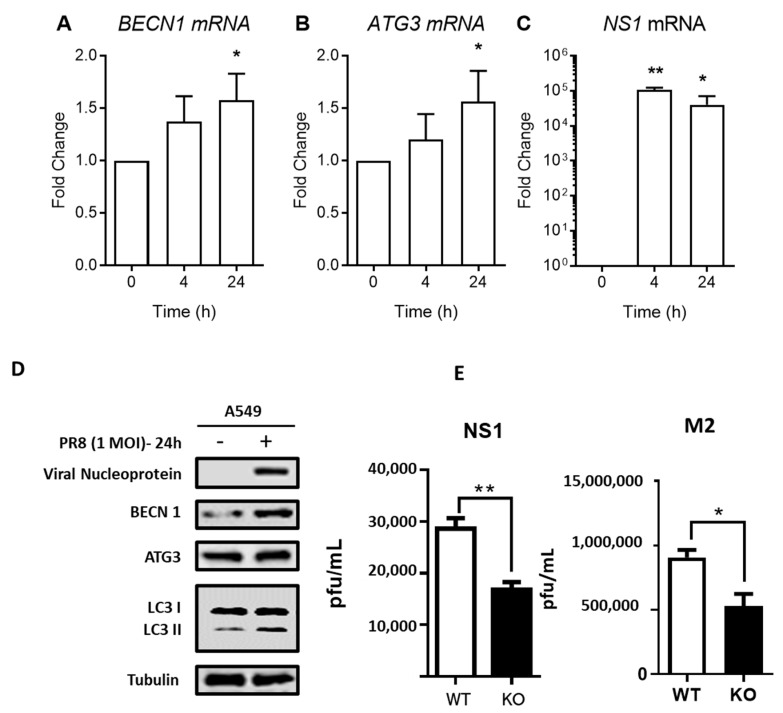
IAV infection increases autophagy-related genes in lung epithelial cells. (**A**–**C**) A549 lung epithelial cells were infected with 1 MOI of PR8 and reverse transcription PCR (RT-PCR) was performed for mRNA analysis of autophagy-related genes BECLN1, ATG3 and virus protein gene non-structural protein 1 (NS1). Glyceraldehyde 3-phosphate dehydrogenase (GAPDH) was used as an internal control for all quantitative RT-PCR analyses. Data are representative of 3–6 SEM of lung epithelial cells. * *p* < 0.05, ** *p* < 0.01, against uninfected cells. (**D**) Western blot analysis of autophagy-related proteins BECN1, ATG3 and LC3-II after IAV infection in A549 cells. (**E**) WT MEF and Atg5^−/−^ MEF cells were infected with 1 MOI of H1N1 influenza. Total RNA was isolated from infected cells at 12 h post-infection. The mRNA level of viral NS1 and M2 were determined by using real-time CR. Results are representative of three independent experiments.

We first validated the role of autophagy in influenza virus infection. The transcription of autophagy-related genes Beclin-1 (BECN1, ATG6) and Autophagy-related gene 3 (ATG3) were determined after IAV infection at 4 and 24 h in A549 lung epithelial cells. BECN1 and ATG3 mRNA were significantly increased after 24 h post-infection (hpi) as compared to their respective uninfected controls (Figure 4A,B). Successful viral infection can be observed from the significant increase in non-structural protein 1 (NS1) viral mRNA expression after infection (Figure 4C). Autophagy-related proteins BECN1 and ATG3 were similarly higher 24 h after IAV infection, together with an increase in LC3-II (Figure 4D), indicating an induction of autophagy after IAV infection. To study the role of autophagy deficiency on the viral replication, the effect of ATG5 deficiency on gene expression of NS1 and M2 were examined using ATG5 WT and ATG5^−/−^ MEFs. After 24 h of infection, both NS1 and M2 gene expression were significantly lower in ATG5^−/−^ MEFs compared to WT MEFs (Figure 4E), suggesting that autophagy is important for viral RNA synthesis, although ATG5-deficient cells may also undergo more apoptosis. Taken together, these results indicate that autophagy is important for influenza virus infection and viral RNA synthesis.

### 3.4. Silencing ANXA1 Results in the Reduction of Autophagosomes and Autophagy after Starvation and Inhibition of mTOR

**Figure 5:**
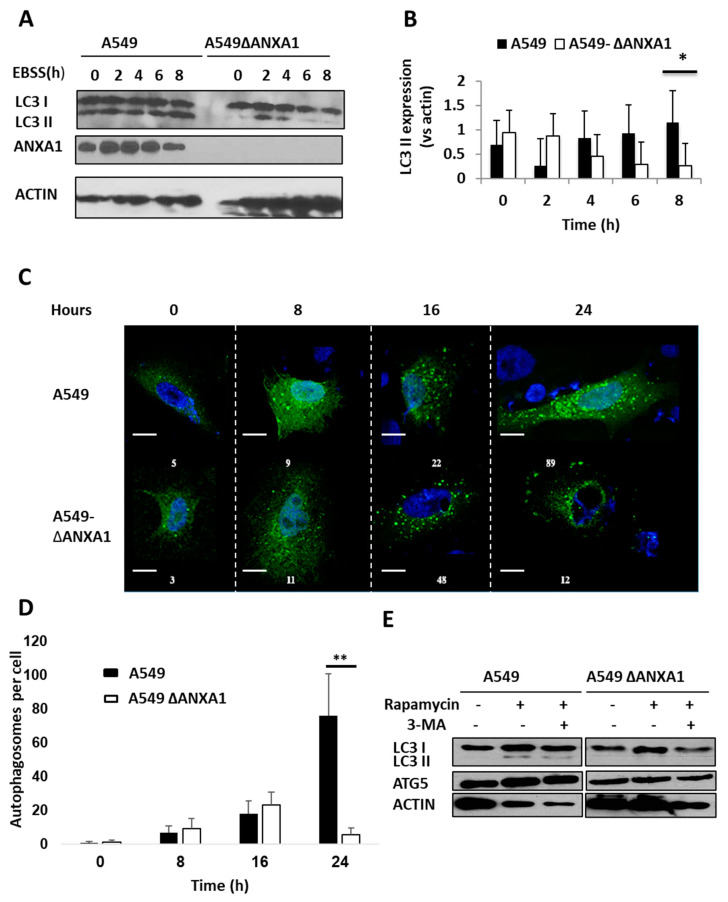
mTOR inhibitors and starvation induce autophagy in A549 but not in A549-ΔANXA1. (**A**,**B**) A549 cells and A549-ΔANXA1 cells were treated with EBSS over various time points (0, 2, 4, 6 and 8 h). The cell lysates were then collected and a Western blot analysis using LC3 antibody was performed. (**C**,**D**) The number of autophagosomes (labeled by anti-LC3-GFP) was counted in A549 cells and A549-ΔANXA1 cells after being treated with EBSS at various time points (0, 8, 16 and 24 h). The autophagosomes were labelled using anti-LC3-GFP (green) and the nuclei of the cells were stained using DAPI (blue). Autophagosomes were counted per cell in at least 3–5 cells per view, and the error bars and significance data were generated from 3 fields of view. (**E**) Western blot analysis was performed after cells were treated with 200 nM of rapamycin and 0.75 mg/mL of 3-Methyladenine (3-MA) for 24 h. Data is representative of three independent experiments. * 0.01 < *p* < 0.05, ** *p* < 0.01. Scale bars = 25 µm in all panels.

Starvation or low nutrient levels triggers autophagy via various signaling pathways, including Tuberous sclerosis 1 (TSC1)-mTOR, recombination-activating genes (RAGs) and AMP-activated protein kinase (AMPK) pathways [31]. Our previous studies demonstrate that ANXA1 is important in IAV infection and apoptosis [21]. Thus, to determine if ANXA1 is important in IAV-dependent autophagy, A549 parental and A549-ΔANXA1 cells were first treated with EBSS media to induce starvation and autophagy for increasing time points. The expression of LC3II was observed to be significantly inhibited in A549-ΔANXA1 cells at the later time points when treated with EBSS (Figure 5A,B).

In addition, confocal staining was performed to detect autophagosomes labelled by anti-LC3 green fluorescent protein (GFP) formed with a long period of starvation. As illustrated in Figure 5C, autophagosome formation was increased in A549 cells but not A549-ΔANXA1 cells after 24 h, with a significant difference in the number of autophagosomes formed per cell (Figure 5D). This suggests that ANXA1 may be important in the regulation of autophagosome formation. MTOR negatively regulates the ULK1 kinase complex and thus inhibits autophagy [28,29]. Therefore, rapamycin, an inhibitor of mTOR [30,31], and Torin-1 (Supplementary Figure S3A,B) were used to induce autophagy. In addition, an autophagy inhibitor, 3-Methyladenine (3-MA), was used together with rapamycin. 3-MA inhibits class III PI3K and thus inhibits autophagy by blocking autophagosome formation (Figure 5E). After 24 h, LC3-II was only expressed in A549 WT cells treated with rapamycin alone and rapamycin with 3-MA, or Torin-1. However, no LC3-II expression was detected in A549-ΔANXA1 cells treated with the same stimuli, indicating that the mTOR inhibitor-induced autophagic flux may be ANXA1-dependent. 

### 3.5. ANXA1 Is Involved in Autophagy Induced by Influenza Virus In Vitro and In Vivo

**Figure 6:**
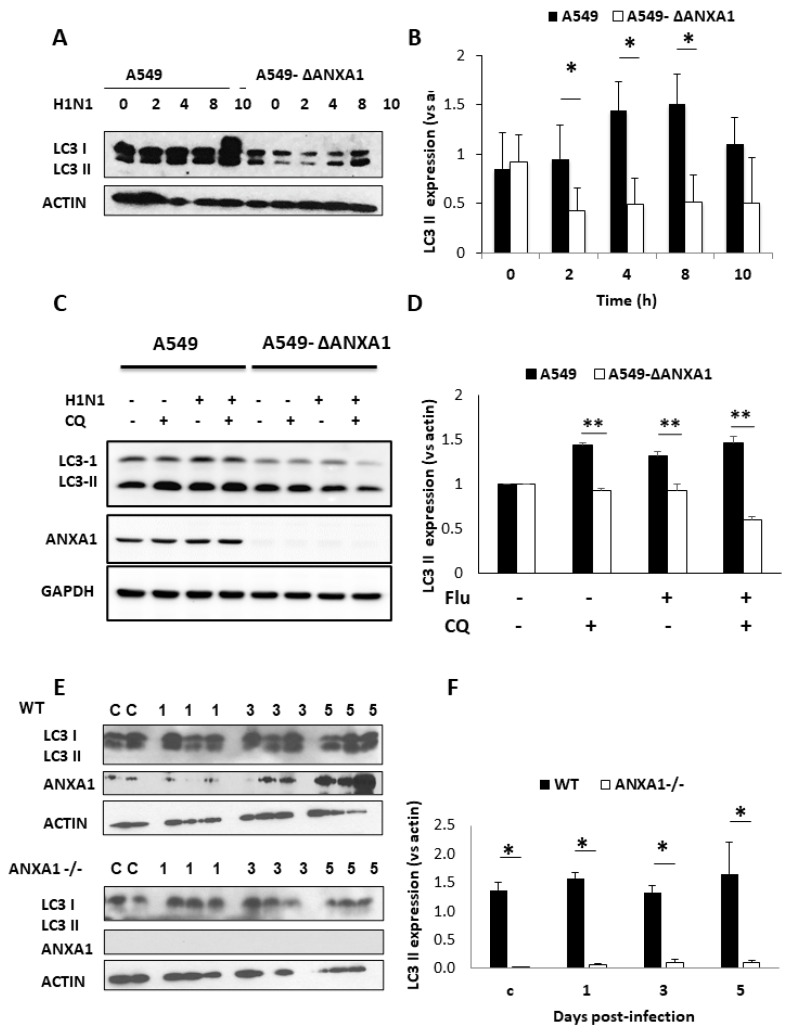
ANXA1 is involved in autophagy induced by influenza virus. (**A**,**B**) Cells were infected with H1N1 at 1 MOI over various time points (0, 2, 4, 8 and 10 h). Quantification of LC3-II expression using densitometric analysis. Data represent two independent experiments (*p* < 0.05). (**C**,**D**) A549 and A549-ΔANXA1 cells were infected with H1N1 at 1 MOI over 12 h, CQ 50 uM for 2 h. Quantification of LC3-II expression using densitometric analysis ** *p* < 0.01. (**E**, **F**) WT and ANXA1^−/−^ Balb/c mice were infected with 500 pfu of influenza virus intratracheally. On days 0, 1, 3 and 5 post-infection, the mice were sacrificed, and their lung lysates were obtained for Western blot analysis. * 0.01 < *p* < 0.05.

Next, to investigate if ANXA1 plays a role in the intracellular degradation process induced by IAV infection, both A549 cells and A549-ΔANXA1 cells were infected with H1N1 PR8 IAV at multiplicity of infection (MOI) 1 over various time points. Upon treatment with IAV, LC3-II expression increases and is significantly higher in A549 cells but not A549-ΔANXA1 cells after 8 h of infection (Figure 6A,B). In addition, to further investigate the role of ANXA1 in IAV-induced autophagy flux, chloroquine (CQ) was used to treat the A549 and A549-ΔANXA1 lung cancer cells with or without IAV infection. IAV infection increased the autophagy flux in A549 cells but not in A549-ΔANXA1 cells, indicating that deletion of ANXA1 can suppress IAV-induced autophagic flux (Figure 6C,D). 

Having shown that ANXA1 plays a positive role in influenza virus-induced autophagy in vitro, we next replicated the results in vivo. WT mice and ANXA1^−/−^ mice were infected with influenza virus at 500 plaque-forming units (pfu). At day 1, day 3 and day 5 post-infection, these mice were sacrificed, and lung lysates were obtained and used to determine the expression of LC3-II after IAV infection. As illustrated in Figure 6E,F, the expression of LC3-II appeared prominently only for WT mice and very little was observed for ANXA1^−/−^ mice, indicating that ANXA1 is indeed required to induce autophagy triggered by IAV. 

In conclusion, our results show that ANXA1 can enhance autophagy induced by conventional autophagy inducers as well as influenza virus.

## 4. Discussion

In this study, transcriptomic RNA-Seq was used to investigate the role of ANXA1 in IAV infection and validated experiments show that ANXA1 is important in classical and IAV-induced autophagy. The abundance and significant over-representation of various transcripts were found in cells expressing and silenced for ANXA1 using CRISPR/Cas9. Our results show unique genes which require ANXA1 to be expressed (WT unique up), genes which require ANXA1 to be suppressed during IAV infection (WT unique down), as well as genes which can be regulated positively (KO unique down) or negatively (KO unique up) by ANXA1, but where ANXA1 is not critical for expression. The highest hit for genes requiring ANXA1 to be expressed belong to pathways related to “immunity and virus response”, including innate immune response and responses to the virus and anti-viral defense. In contrast, genes requiring ANXA1 to be suppressed during IAV are those shown to be involved in viral transcription and translation, including genes which are highly enriched in translational regulation, such as RPS3, RPL23, RPL24 and RPL25. These are ribosomal proteins involved in influenza virus RNA transcription and viral mRNA translation. Unique genes upregulated after IAV infection in A549-∆ANXA1 cells but not in WT A549 cells, which are genes which can be negatively regulated by ANXA1, are enriched mostly in mitochondria and mitochondrial function. This includes enzymes such as HAGH (Hydroxyacyl glutathione Hydrolase), POLRMT (RNA polymerase in Mitochondria), ACSM4 (Acyl-CoA Synthetase), LIAS (Lipoic Acid Synthetase) and COX7A (Cytochrome c oxidase). This suggests that ANXA1 can regulate many enzymes involved in lipid metabolism and mitochondrial metabolism. This demonstrates a multi-pronged function of ANXA1, where ANXA1 is required for the expression of anti-viral genes in response to type-1 interferons, similar to what has been described previously [32]. In contrast, genes requiring ANXA1 to be suppressed during IAV are those shown to be involved in viral transcription and translation. This demonstrates a multi-pronged function of ANXA1, where ANXA1 is required for the expression of anti-viral genes in response to type-1 interferons, yet is also required for the suppression of genes important in virus transcription and translation. ANXA1 also is required for the expression of genes relating to endosomal trafficking and autophagosome assembly, which can be linked to our previous work, showing that ANXA1 promotes virus replication through enhancement of endosomal trafficking [21]. Genes which ANXA1 can positively regulate are centered around cell adhesion and neuronal development, while genes which ANXA1 can negatively regulate are mostly related to mitochondrial function and cell metabolism. As ANXA1 has been shown to enhance virus replication in vitro and in vivo [21], it may be possible to predict that the roles of ANXA1 in endosomal trafficking and the regulation of mitochondrial function may have more impact on virus replication than the regulation of anti-viral immune responses.

While autophagy is shown to promote IAV replication and apoptosis [33,34] and various viral proteins such as nucleoprotein (NP) and M2 can induce the AKT-mTOR autophagy pathway [35], M2 has also been shown to stimulate the initial phase of autophagosome formation, but inhibits autophagosome–lysosome fusion, resulting in the inhibition of anti-apoptotic macroautophagy, a strategy to enhance its pathogenicity [3]. Enhanced host cell death could limit virus-specific host responses and cytokine production. Whether the stimulation of autophagy is a host-response to viral infection, or the strategy of the virus against the host itself, is yet to be determined. Nevertheless, autophagy and autophagic cell death is essential in influenza replication and pathogenesis. In addition, annexin–pathogen interactions have been well elaborated recently and show that IAV utilize ANXA1 to regulate the host innate immune responses [21,36]. We recently showed that ANXA1 enhances influenza virus infection and viral replication by enhancing cell death and apoptosis [21]. Using RNA-Seq and in vitro and in vivo validation, our study establishes that ANXA1 can enhance autophagy and is crucial for influenza virus-induced autophagy. However, this is not in line with a recent study which demonstrated that silencing of ANXA1 with siRNA significantly inhibited starvation-induced autophagic degradation as measured by the level of Sequestosome 1 (SQSTM1 or p62), although the specific siRNA did not alter starvation-induced LC3-II level [37]. In our study, the expression of LC3-II was higher in A549 parental cells compared to A549-ΔANXA1 cells when treated with EBSS and other autophagy inducers. This discrepancy may be due to the fully functional silencing of ANXA1 via CRISPR-Cas9, or cell-type specific differences. These results suggest that the presence of ANXA1 is important in autophagy triggered by starvation, mTOR inhibitors and influenza virus. 

Overall, the data in this study establishes the positive role of ANXA1 in virus-induced autophagy as well as autophagy triggered by other mechanisms that include starvation or inhibitors of mTOR. Hence, the presence of ANXA1 directly benefits viral replication through the enhancement of autophagy, another mechanism through which ANXA1 can act to enhance virus propagation.

## Supplementary Materials

The following are available online at https://www.mdpi.com/2073-4409/9/6/1399/s1: Figure S1: Torin 1 induces autophagy in a time-dependent manner in A549 but not in A549. Figure S2: Pathways involved in the response caused by IAV infection in A549 (WT) and A549 ΔANXA1 (KO) infected cells. Figure S3: Gene Set Enrichment Analysis (GSEA) analysis in IAV infected A549 WT and A549 ΔANXA1 cells. Table S1: Off-target effects of #4 CRISPR plasmid, Table S2: Differentially expressed genes, Table S3: Gene Set Enrichment Analysis.
