# Supplementary material for: RNA-Sequencing-Based Transcriptomic Analysis Reveals a Role for Annexin-A1 in Classical and Influenza A Virus-Induced Autophagy
Source: Cells. 2020 Jun 4;9(6):1399. doi: 10.3390/cells9061399 (PMC7349256; doi:10.3390/cells9061399)
Supplement: Supplementary file 1 [file cells-09-01399-s001.zip › cells-804836-revised supplementary/cells-804836 -Table S1 off target.pdf]

Result	Query tag	Search result	Query type	Mismatch	Ends with RG	Chr Position	Strand	Cut site	Score
ATGCAAGGCAGCGACATCCGAGG -- hit	Query:	hit:							
ATGCAAGGCAGCGACATCCGNGG -- query	ATGCAAGGCAGCGACATCCGNGG	ATGCAAGGCAGCGACATCCGAGG	No indel	0	Yes	Chr9:73158762-73158784	-	73158768	0
TGCAAGGCAGCGACATCCGAGG -- hit	Query:	hit:							
TGCAAGGCAGCGACATCCGNGG -- query	TGCAAGGCAGCGACATCCGNGG	TGCAAGGCAGCGACATCCGAGG	Del 20	0	Yes	Chr9:73158762-73158783	-	73158768	0.63
TGCAAGGCAGCGACATCCGAGG -- hit	Query:	hit:							
AGCAAGGCAGCGACATCCGNGG -- query	AGCAAGGCAGCGACATCCGNGG	TGCAAGGCAGCGACATCCGAGG	Del 19	1	Yes	Chr9:73158762-73158783	-	73158768	0.76
ATGCAAGGCAGCGACATCCGAG -- hit	Query:	hit:							
ATGCAAGGCAGCGACATCCNGG -- query	ATGCAAGGCAGCGACATCCNGG	ATGCAAGGCAGCGACATCCGAG	Del 1	1	Yes	Chr9:73158763-73158784	-	73158769	26.51
ATGCAAGGCAGCGACATCCGAG -- hit	Query:	hit:							
ATGCAAGGCAGCGACATCCGGG -- query	ATGCAAGGCAGCGACATCCGGG	ATGCAAGGCAGCGACATCCGAG	Del PAM 3	1	Yes	Chr9:73158763-73158784	-	73158769	40.51
ATGCAAGGCAGCGACATCCGAG -- hit	Query:	hit:	Del PAM 1, or Del						
ATGCAAGGCAGCGACATCCGNG -- query	ATGCAAGGCAGCGACATCCGNG	ATGCAAGGCAGCGACATCCGAG	PAM 2	0	Yes	Chr9:73158763-73158784	-	73158769	20.51
TATGCAAGGCAGCGACATCCGAGG -- hit	Query:	hit:							
ANTGCAAGGCAGCGACATCCGNGG -- query	ANTGCAAGGCAGCGACATCCGNGG	TATGCAAGGCAGCGACATCCGAGG	Ins 19	1	Yes	Chr9:73158762-73158785	-	73158768	0.95
ATGCAAGGCAGCGACATCCGAGGA -- hit	Query:	hit:	Ins PAM 2, or Ins						
ATGCAAGGCAGCGACATCCGNNGG -- query	ATGCAAGGCAGCGACATCCGNNGG	ATGCAAGGCAGCGACATCCGAGGA	PAM 3	1	No	Chr9:73158761-73158784	-	73158767	40.7
ATGCAAGGCAGCGACATCCGAGGA -- hit	Query:	hit:							
ATGCAAGGCAGCGACATCCGNGNG -- query	ATGCAAGGCAGCGACATCCGNGNG	ATGCAAGGCAGCGACATCCGAGGA	Ins PAM 1	1	No	Chr9:73158761-73158784	-	73158767	40.7
