# Supplementary material for: RNA-Sequencing-Based Transcriptomic Analysis Reveals a Role for Annexin-A1 in Classical and Influenza A Virus-Induced Autophagy
Source: Cells. 2020 Jun 4;9(6):1399. doi: 10.3390/cells9061399 (PMC7349256; doi:10.3390/cells9061399)
Supplement: Supplementary file 1 [file cells-09-01399-s001.zip › cells-804836-revised supplementary/cells-804836-supplementary figures.pdf]

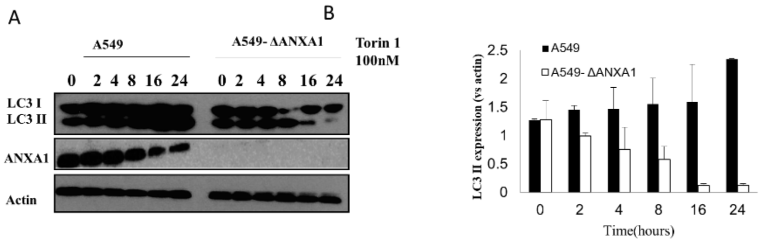

Figure S1. Torin 1 induces autophagy in a time-dependent manner in A549 but not in A549-ΔANXA1. (**A**) A549 cells were treated with Torin 1 over various time points (0, 2, 4, 8, 16 and 24 h). The cells lysates were then collected and a western blot analysis using LC3 and ANXA1 antibody was performed. (**B**) Quantification of LC3 II expression is shown using image J (https://imagej.nih.gov/ij/). Data is a representative of three independent experiments.

WT UP

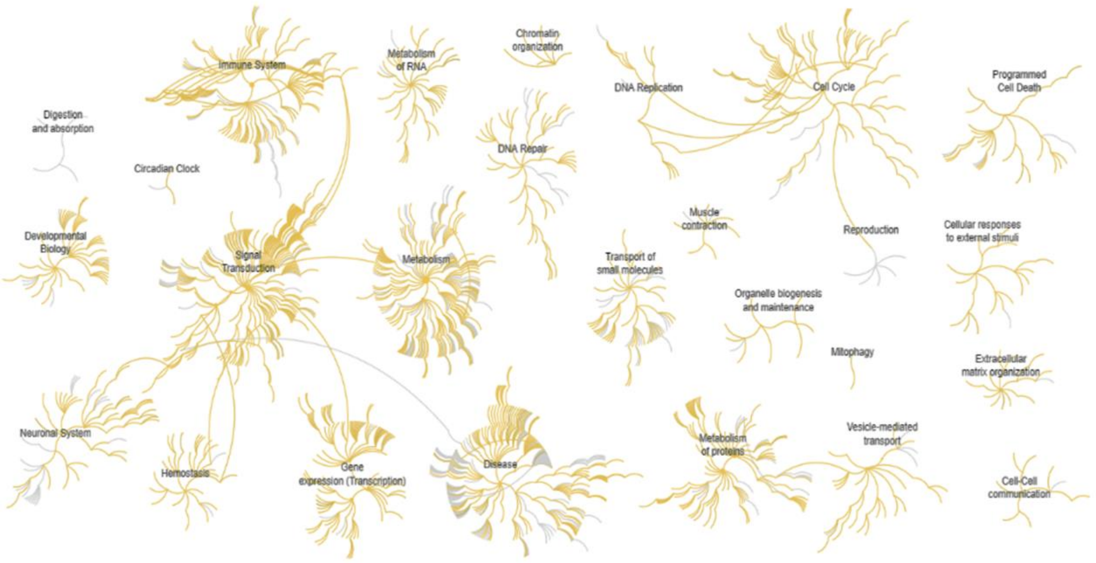

Immune System
Metabolism of RNA
Chromatin organization
DNA Replication
Cell Cycle
Programmed Cell Death
Digestion and absorption
Circadian Clock
DNA Repair
Muscle contraction
Reproduction
Cellular responses to external stimuli
Developmental Biology
Signal Transduction
Metabolism
Transport of small molecules
Organelle biogenesis and maintenance
Mitophagy
Extracellular matrix organization
Neuronal System
Hemostasis
Gene expression (Transcription)
Disease
Metabolism of proteins
Vesicle-mediated transport
Cell-Cell communication

WT DOWN

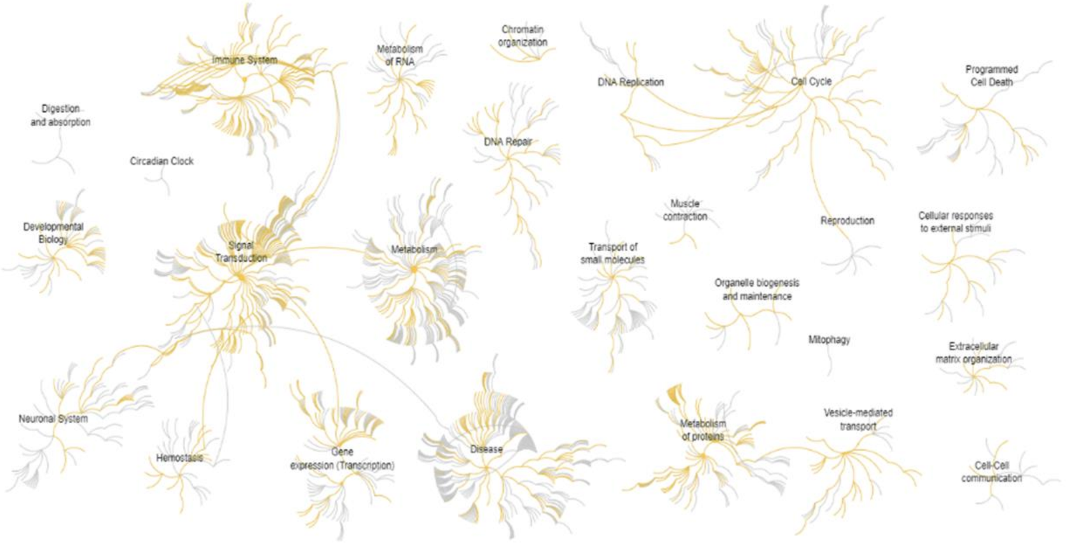

Immune System
Metabolism of RNA
Chromatin organization
DNA Replication
Cell Cycle
Programmed Cell Death
Digestion and absorption
Circadian Clock
DNA Repair
Muscle contraction
Reproduction
Cellular responses to external stimuli
Developmental Biology
Signal Transduction
Metabolism
Transport of small molecules
Organelle biogenesis and maintenance
Mitophagy
Extracellular matrix organization
Neuronal System
Hemostasis
Gene expression (Transcription)
Disease
Metabolism of proteins
Vesicle-mediated transport
Cell-Cell communication

KO DOWN

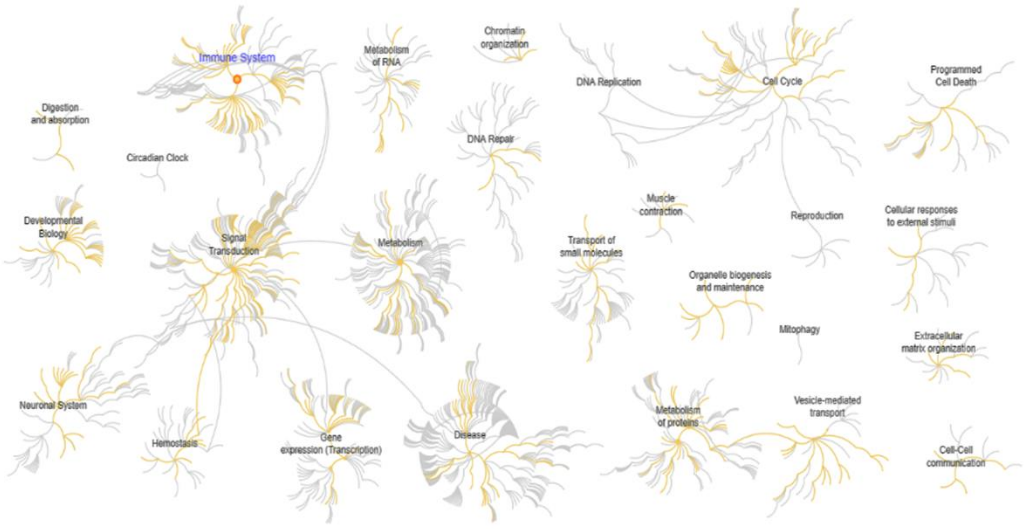

KO UP

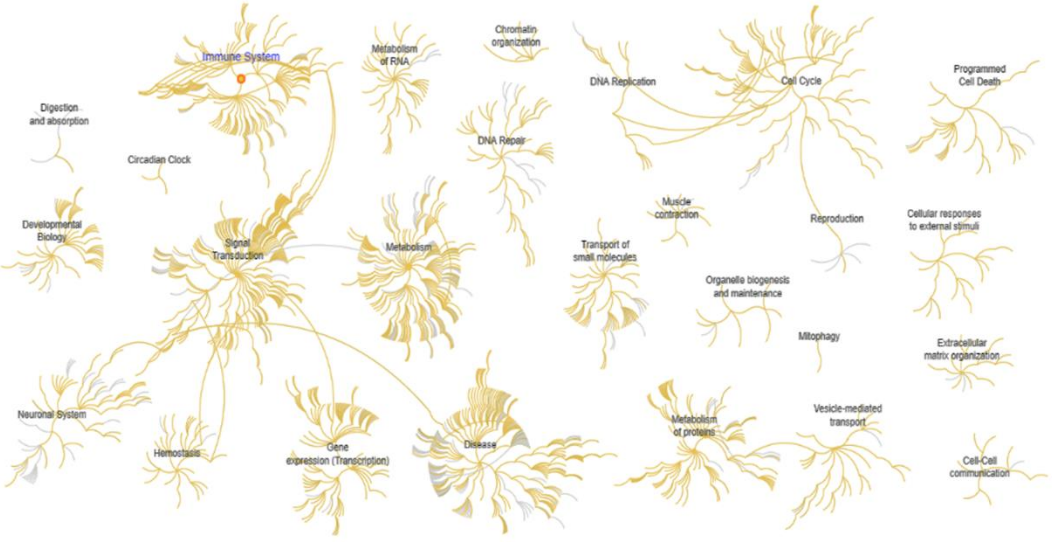

Figure S2. Pathways involved in the response caused by IAV infection in A549 (WT) and A549ΔANXA1(KO) infected cells. A genome-wide overview of the results of pathway analysis for 4 groups. The center of each of the circular "bursts" is the root of one top level pathway. Each step away from the center represents the next level lower in the pathway hierarchy. The color code denotes over-representation of that pathway in the input dataset. Light grey signifies pathways which are not significantly over-represented.

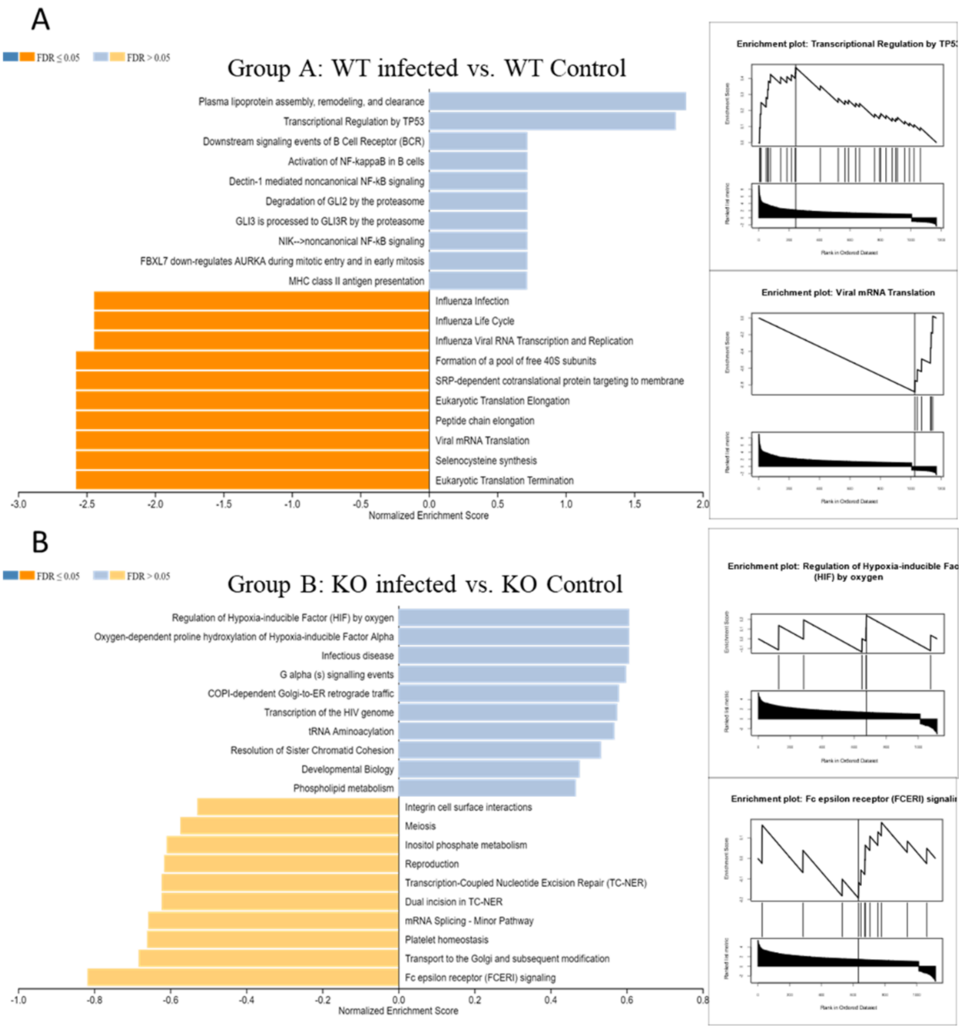

Figure S3. Gene Set Enrichment Analysis (GSEA) analysis in IAV infected A549 WT and A549 ΔANXA1 cells. 10 positive related categories and 10 negative related categories are identified as enriched categories in the bar chart and selected enrichment plot are shown. (**A**) Significant enriched pathways of the highly expressed DEGs in upregulation and downregulation pattern in group A (WT infected vs. WT Control). (**B**) Significant enriched pathways of the highly expressed DEGs in upregulation and downregulation PATTERN in group B (KO infected vs. KO CONTROL).
